# The association between urbanization and reduced renal function: findings from the China Health and Nutrition Survey

**DOI:** 10.1186/s12882-017-0577-7

**Published:** 2017-05-15

**Authors:** Yosuke Inoue, Annie Green Howard, Amanda L. Thompson, Michelle A. Mendez, Amy H. Herring, Penny Gordon-Larsen

**Affiliations:** 1 Carolina Population Center, The University of North Carolina at Chapel Hill, Chapel Hill, NC 27516 USA; 2 Department of Biostatistics, Gillings School of Global Public Health, The University of North Carolina at Chapel Hill, Chapel Hill, NC 27599 USA; 3 Department of Anthropology, The University of North Carolina at Chapel Hill, Chapel Hill, NC 27599 USA; 4 Department of Nutrition, Gillings School of Global Public Health, The University of North Carolina at Chapel Hill, Chapel Hill, NC 27599 USA

**Keywords:** Glomerular filtration rate, Creatinine, Renal insufficiency, China, Urbanization

## Abstract

**Background:**

While chronic kidney disease (CKD) is a growing public health concern in low- and middle-income countries, such as China, few studies have investigated the association between urbanization and the occurrence of CKD in those countries.

**Methods:**

We investigated the association between urbanization and estimated glomerular filtration rate (eGFR), an important CKD risk marker. Data came from the China Health and Nutrition Survey wave 2009, in which we collected fasting serum, individual and household data along with community level urbanization data, which was used to derive a study-specific urbanization measure, in 218 communities across nine provinces. A total of 3644 men and 4154 women participants aged 18 years or older were included in the analysis. Reduced renal function was defined as eGFR of less than 60 mL/min/1.73 m^2^ measured using serum creatinine concentration (mg/dL).

**Results:**

After adjusting for socio-demographic (e.g., age, education and household income), a sex-stratified multilevel logistic model revealed that living in a more urbanized community was associated with higher odds of reduced eGFR (odds ratio [OR] = 1.38 per one-standard deviation [SD] increase in the CHNS specific urbanization index, 95% confidence interval [CI] = 1.11–1.73 for men; OR = 1.35, 95% CI = 1.11–1.62 for women). After adjusting for behavioral variables (i.e., alcohol consumption, smoking, physical activity and diet), as well as obesity and cardiometabolic risk factors, the association was attenuated in men (OR = 1.25, 95% CI = 0.98–1.59), but remained statistically significant in women (OR = 1.24, 95% CI = 1.01–1.52).

**Conclusion:**

Our findings suggest that living in an urban environment is linked with higher odds of reduced renal function independently of behavioral and cardiometabolic risk factors, which have been shown to increase along with urbanization.

**Electronic supplementary material:**

The online version of this article (doi:10.1186/s12882-017-0577-7) contains supplementary material, which is available to authorized users.

## Background

Chronic kidney disease (CKD) is an important risk factor for end-stage renal disease [1], all-cause and cardiovascular mortality [2], and non-vascular outcomes (e.g., cognitive decline and functional impairment [3, 4]). Lozano et al. [5] reported an increase in CKD of 15% between 1990 and 2010 across the globe. Individuals in low- and middle-income countries were disproportionally affected [6] perhaps due to increases in non-communicable disease and exposure to environmental toxins along with urbanization [7]. In addition, older age [3, 8–14], being a woman [3, 8, 10, 11, 14, 15], having metabolic syndromes [3, 8–14, 16–18], smoking [9, 10, 17–19] or lower socioeconomic status (SES) [3, 20] have been shown to be associated with development and progression of CKD.

Despite the potential influence on some of the above-mentioned CKD risk factors, relatively few studies have examined community context in relation to the development of CKD. In the USA and the UK, living in a low SES community has been linked with higher risk of CKD [21, 22]. In developing countries, on the other hand, there have been conflicting results. For example, Wang et al. [23] showed that CKD prevalence was higher in urban areas (where SES was higher on average) compared with rural areas. Using Chinese data in 2011 to 2012. Conversely, Zhang et al. [14] showed no statistically significant difference between urban and rural residents in China in 2009 to 2010 and Kaze et al. [12] reported that the prevalence of CKD was higher in rural areas compared to in urban areas in Cameroon.

As renal replacement therapy is not always available to CKD patients in developing countries where economic and medical resources are scant [7, 24], there is an urgent need to identify those at higher risk of CKD at an earlier stage. It is also critical to identify correlates of CKD that could be candidates for preventive efforts, while distinguishing the contribution attributed to individual behavioral risk factors versus community-level factors, which may require different intervention strategies. For example, discrepant findings for CKD in rural versus urban areas may relate to use of a conventional dichotomous administrative classification of urban versus rural. Yet, urbanization captures several nuanced aspects of the community environment [25]. Second, few studies have used multilevel models to estimate contextual effects (i.e., residents in the same community tend to experience a similar exposure and health status [26]). Third, few studies have investigated the role of diet and physical activity behaviors in relation to the association between urbanization and CKD in developing countries.

To address these gaps, we used a validated urbanization index assessed at community level and estimated glomerular filtration rate (eGFR) to investigate the association between urbanization and reduced renal function in China. We used data from the China Health and Nutrition Survey (CHNS) and employed a multilevel model controlling for socio-demographic (i.e., age, education and household income) and behavioral variables (i.e., alcohol consumption, smoking, physical activity and diet), body mass index (BMI) and cardio-metabolic factors (i.e., blood pressure, hemoglobin A1c, fasting blood glucose and low-density lipoprotein) to examine the association between urbanization and CKD.

## Methods

### China Health and Nutrition Survey

The CHNS is an ongoing longitudinal study which collected data from 228 communities across nine provinces (i.e., Heilongjiang, Liaoning, Shandong, Henan, Jiangsu, Hunan, Hubei, Guangxi, and Guizhou). Multistage random-cluster design and a stratified probability sample were used to select counties and cities stratified by income using State Statistical Office definitions [27]; we then selected participating communities and households from these strata. The cohort mirrored national profiles in relation to age, sex and education initially [28–30] and the provinces in the CHNS sample comprised 44% of the population in China as of 2009. Details on the CHNS procedures have been described previously [31].

Fasting blood was available in wave 2009 data to generate serum creatinine data for 9493 participants aged 18 years or older. After excluding those who did not have information on age (*n* = 5), education (*n* = 54), household income (*n* = 133), health-related behaviors (*n* = 1303) and the other biomarkers (*n* = 200), we have an analytic sample of 7798 participants (3644 men and 4154 women) living in 218 communities.

### Dependent variable

Using fasting serum to derive serum creatinine concentration (Scr, mg/dL), we calculated estimated glomerular filtration rates (eGFR), using the following set of equations [32] which were modified based on the Chronic Kidney Disease-Epidemiology (CKD-EPI) equations and acquired the best performance among serum creatinine-based equations validated among Chinese population in Ye et al. [33]. Reduced renal function was defined as eGFR of <60 mL/min/1.73 m^2^ [34].

**Table:** 

Woman	Scr ≤ 7 mg/dL	144 × (Scr/0.7)^0.156^ × 0.993^age^;
	Scr > 7 mg/dL	144 × (Scr/0.7)^-1.057^ × 0.993^age^;
Man	Scr ≤ 9 mg/dL	141 × (Scr/0.9)^0.074^ × 0.993^age^;
	Scr > 9 mg/dL	141 × (Scr/0.9)^-1.057^ × 0.993^age^

### Explanatory variables

We used a 12-component study-specific urbanization index, which was previously validated to capture the degree of urbanization in the study communities (reliability across study waves [Cronbach’s Alpha]: 0.85–0.89; validity [correlation with official classification]; 0.75–0.78) [25]. The following 12 components were included in the development of the urbanization index: (1) population density; (2) types of economic activity; (3) traditional market; (4) modern markets; (5) transportation and infrastructure; (6) sanitation; (7) communications (e.g., TV, mobile, post, and cinema); (8) housing (e.g., electricity, indoor tap water, and flush toilet); (9) education; (10) diversity (i.e., variation in community education level and variation in community income level); (11) health infrastructure; and (12) social services. The scale has a possible range of 0–120, with a higher score reflecting more urban characteristics across these twelve multiple domains.

Sociodemographic and behavior data obtained using the CHNS questionnaire included age (in years), sex (men and women), education (graduated from primary school or less; graduated from junior high school; graduated from high school; and attained further education), annual household income per capita tertiles (0–6533 yuan [low]; 6542–13,859 yuan [middle]; and 13,862–378,571 yuan [high]), health-related behaviors such as alcohol (< / ≥ once per month), current smoking (yes, no), and weekly physical activity level derived from a detailed seven-day PA recall across a variety of domains. We also used diet data derived from three consecutive 24-h dietary recalls with energy and nutrient content of foods estimated based on a Chinese food composition table [35]. This dietary assessment has been validated relative to doubly labeled water for energy (r: 0.56 [men] and 0.60 [women]) [36] and urine for sodium (r: 0.58) and potassium (r: 0.59) [37]. We included protein consumption and energy intake and checked changes in the results by including either sodium intake and potassium intake or sodium-to-potassium ratio. To investigate the impact of non-communicable diseases as potential mediators linking urbanization and CKD, we used anthropometric data to classify overweight and obesity (overweight: body mass index [BMI; kg/m^2^]: 24.0–27.99; obesity: BMI ≥ 28.0 [38]) and hypertension (either systolic pressure ≥ 140 mmHg, diastolic pressure ≥ 90 mmHg or self-reported antihypertensive medication [39]) and fasting blood used to classify diabetes mellitus (DM) (either hemoglobin A1c ≥ 6.5% or fasting blood glucose ≥126 mg/dL or self-reported diagnosis of DM [39]) and high concentration of low-density lipoprotein cholesterol (LDL; ≥ 130 mg/dL [39]).

### Statistical analyses

Descriptive characteristics of the study participants were compared by eGFR status (i.e., < / ≥ 60 mL/min/1.73 m^2^) using either t-tests (for continuous variables) or χ^2^ tests (for categorical variables) in each sex. A sex-stratified multilevel model was used to investigate the association between the community-level urbanization index and reduced renal function while accounting for multiple individuals within a community with a random intercept for individual. We used several steps for adjustment; specifically, the initial model included age, sex, educational attainment and household income (Model 1). Then we added health-related behaviors: frequency of alcohol consumption (< / ≥ once per month), current smoking (men only due to small numbers of women smokers) (yes, no), physical activity (per 10 metabolic equivalents [METs]), energy intake (per 1000 kcal), protein intake (per 100 g), sodium intake and potassium intake in Model 2 or sodium-to-potassium ratio in Model 3. We then fuether adjusted Model 2 for BMI and cardiometabolic risk factors (hypertension, DM, and high LDL cholesterol) in Model 4. Overweight and obesity were combined due to small sample sizes.

In addition, to better understand the pathways linking urbanization and reduced renal function, we additionally conducted a separate analysis in which we incorporated the 12 components of the urbanization index in the model while adjusting for the covariates included in Model 4 and also stratifying by sex.

All statistical analyses were conducted using Stata 14.0 (College Station, TX). The level of statistical significance (two-tailed) was set at *p* < 0.05.

## Results

Table 1 shows the basic characteristics of the study participants. The prevalence of reduced renal function was 8.1% (6.4% for men and 9.6% for women). Those with reduced renal function tended to be considerably older than those without (71.5 years vs 49.6 years, *p* < 0.001 for men; 69.4 years vs. 49.0 years, *p* < 0.001 for women), were more likely to have a lower educational attainment (i.e., graduation from primary school or less; 54.5% vs. 33.1%, *p* < 0.001 for men; 80.7% vs. 49.0%, *p* < 0.001 for women), and were more likely to have low household income (33.5% vs. 31.9%, *p* = 0.049 for men; 38.1% vs. 34.2%, *p* < 0.001 for women). In terms of numerous health-related behaviors, individuals with reduced renal function differed significantly from others with markedly lower levels of alcohol consumption (among men; *p* < 0.001), smoking (among men; *p* < 0.001), physical activity (*p* < 0.001), energy intake (*p* < 0.001), protein intake (*p* < 0.001), sodium intake (*p* = 0.009 for men; *p* = 0.004 for women), and potassium intake (*p* < 0.001). Furthermore, those with reduced renal function were more likely to have hypertension (64.4% vs 31.6%, *p* < 0.001 for men; 56.4% vs. 25.9, *p* < 0.001 for women), DM (21.0% vs. 11.5%, *p* < 0.001 for men and 25.8% vs. 8.6%, *p* < 0.001 for women), and high LDL cholesterol (only among women; 52.4% vs. 31.0%, *p* < 0.001). Those who lived in more urbanized communities tend to be older, more educated, and richer, consume more protein, be more hypertensive while they consumed less alcohol, smoked less, exercised less and consumed less energy. There were no rural-urban gradients in relation to potassium intake, sodium-to-potassium ratio, obesity, diabetes and high LDL (Additional file 1: Table S1).

**Table 1:** Basic characteristics of the study participants in China Health and Nutrition Survey (2009), stratified by sex and estimated glomerular filtration rate

	Men (*n* = 3644)					Women (*n* = 4154)				
	eGFR ≥60 (*n* = 3411)		eGFR <60 (*n* = 233)		*p*-value^a^	eGFR ≥60 (*n* = 3755)		eGFR <60 (*n* = 399)		*p*-value^a^
Age (in years), mean [SD]	49.6	14.4	71.5	9.7	< 0.001	49.0	13.7	69.4	10.7	< 0.001
Education, n (%)										
Primary school or less	1130	33.1	127	54.5	< 0.001	1838	49.0	322	80.7	< 0.001
Junior high school	1327	38.9	53	22.8		1142	30.4	44	11.0	
Senior high school	482	14.1	16	6.9		383	10.2	12	3.0	
Post-secondary education	472	13.8	37	15.9		392	10.4	21	5.3	
Household income (yuan), n (%)										
Low (0–6533)	1087	31.9	78	33.5	0.049	1284	34.2	152	38.1	0.280
Middle (6542–13,859)	1164	34.1	62	26.6		1250	33.3	122	30.6	
High (13,862–378,571)	1160	34.0	93	39.9		1221	32.5	125	31.3	
Health-related behaviors										
Alcohol consumption frequency, n (%)	1883	55.2	67	28.8	< 0.001	223	5.9	20	5.0	0.454
Current Smoking, n (%)	1923	56.4	89	38.2	< 0.001	136	3.6	26	6.5	0.005
Weekly physical activity (METs), mean [SD]	224.1	216.7	81.0	119.6	< 0.001	229.6	215.9	110.8	137.7	< 0.001
Energy intake (kcal), mean [SD]	2335.5	600.3	2048.7	577.0	< 0.001	1996.0	545.9	1793.9	518.3	< 0.001
Protein intake (g), mean [SD]	71.7	22.5	62.5	20.0	< 0.001	61.9	20.3	55.1	19.2	< 0.001
Sodium intake (g), mean [SD]	4.9	2.6	4.4	2.6	0.009	4.6	2.6	4.2	2.4	0.004
Potassium intake (g), mean [SD]	1.7	0.6	1.5	0.6	< 0.001	1.6	0.6	1.4	0.5	< 0.001
Sodium-to-potassium ratio, mean [SD]	3.0	1.9	3.2	2.2	0.266	3.1	2.1	3.3	2.1	0.122
Cardiometabolic risk factors										
BMI, n (%)										
< 24.0	2028	59.5	147	63.1	0.547	2254	60.0	238	59.7	0.245
24.0–27.99	1087	31.9	68	29.2		1108	29.5	109	27.3	
≥ 28.0	296	8.7	18	7.7		393	10.5	52	13.0	
Hypertension, n (%)	1078	31.6	150	64.4	< 0.001	971	25.9	225	56.4	< 0.001
Diabetes mellitus, n (%)	392	11.5	49	21.0	< 0.001	324	8.6	103	25.8	< 0.001
High LDL, n (%)	938	27.5	77	33.1	0.068	1164	31.0	209	52.4	< 0.001
Urbanization index, mean [SD]	66.3	19.4	73.6	19.7	< 0.001	66.7	19.4	72.0	19.2	< 0.001
eGFR, mean [SD]	83.8	18.6	50.7	9.9	-	84.5	13.4	54.1	9.3	-

^a^Characteristics of the participants were compared using t-test for continuous variables and χ^2^ test for categorical variables

Table 2 shows the results of a multilevel logistic regression analysis in men. In Model 1, urbanization was positively associated with the odds of reduced renal function. Specifically, a one-standard deviation increase in the urbanization index (i.e., 19.5) was associated with a 1.38 times higher odds of reduced renal function (95% CI = 1.11–1.73). An additional adjustment for behavioral variables in Model 2 attenuated the association between urbanization and reduced renal function, but the association remained statistically significant (OR = 1.27, 95% CI = 1.00–1.61). Alcohol consumption (OR = 0.54, 95% CI = 0.37–0.77) and weekly physical activity level (OR = 0.99, 95% CI = 0.97–1.00) were each inversely associated with reduced renal function; these associations observed in Model 2 did not change when sodium and potassium intakes were replaced with sodium-to-potassium ratio in Model 3. After adjusting for BMI and the cardiometabolic risk factors (Model 4), the association between urbanization and reduced renal function were attenuated (OR = 1.25, 95% CI = 0.98–1.59) and hypertension (OR = 1.93, 95% CI = 1.35–2.74) was shown to be positively associated with reduced renal function.

**Table 2:** Results of a multilevel logistic model to investigate the association between urbanization and reduced renal function among Chinese men (2009)

	Model 1			Model 2			Model 3			Model 4		
	OR	95% CI	*p*-value	OR	95% CI	*p*-value	OR	95% CI	*p*-value	OR	95% CI	*p*-value
Urbanization^a^	1.38	1.11, 1.73	0.004	1.27	1.00, 1.61	0.048	1.28	1.01, 1.62	0.042	1.25	0.98, 1.59	0.074
Health-related behavior												
Alcohol consumption^b^				0.54	0.37, 0.77	0.001	0.53	0.37, 0.76	0.001	0.52	0.36, 0.75	< 0.001
Smoking^c^				0.86	0.60, 1.22	0.395	0.87	0.61, 1.23	0.420	0.90	0.63, 1.30	0.585
Total physical activity^d^				0.99	0.97, 1.00	0.043	0.99	0.97, 1.00	0.041	0.99	0.98, 1.00	0.084
Energy intake^e^				1.29	0.83, 2.00	0.251	1.18	0.77, 1.82	0.440	1.26	0.81, 1.97	0.308
Protein intake^f^				0.95	0.24, 3.73	0.940	0.47	0.14, 1.60	0.228	0.81	0.20, 3.24	0.760
Sodium intake				0.95	0.89, 1.03	0.199				0.95	0.88, 1.02	0.157
Potassium intake				0.70	0.45, 1.08	0.111				0.72	0.46, 1.12	0.148
Sodium-to-potassium ratio							0.96	0.88, 1.05	0.407			
Cardiometabolic risk factors												
Overweight/Obesity^g^										1.03	0.71, 1.50	0.858
Hypertension^h^										1.93	1.35, 2.74	< 0.001
Diabetes Mellitus^i^										1.36	0.88, 2.11	0.162
High LDL^j^										1.17	0.80, 1.69	0.419

Models were adjusted for age (in years), educational attainment (primary education or less, junior high school, high school and attained further education) and household income (low, middle and high)

^a^Results are expressed per one-SD increase

^b^The referent was those who drank less than once per month

^c^The referent was those who didn’t smoke

^d^Results are expressed per 10 METs increase

^e^Results are expressed per 1000 kcal increase

^f^Results are expressed per 100 g increase

^g^The referent was those whose body mass index was 24.0 or less

^h^Hypertension was defined by either systolic pressure ≥ 140 mmHg, diastolic pressure ≥ 90 mmHg or self-reported antihypertensive medication

^i^Diabetes were defined by either hemoglobin A1c ≥ 6.5% or fasting blood glucose ≥126 mg/dL or self-reported diagnosis of DM

^j^Higher LDL was defined by LDL ≥ 130 mg/dL

Table 3 shows the results of a multilevel logistic regression analysis in women. Urbanization was significantly associated with reduced renal function in all models (e.g., OR = 1.24, 95% CI = 1.01–1.52 in Model 4). In Models 2 and 4, there was an inverse relationship between potassium intake and reduced renal function (e.g., OR = 0.64, 95% CI = 0.44–0.92 in Model 2). The cardiometabolic risk factors investigated in this study were positively associated with reduced renal function; specifically hypertension (OR = 1.34, 95% CI = 1.01–1.78), diabetes mellitus (OR = 2.07, 95% CI = 1.47–2.92), and high low-density lipoprotein (OR = 1.42, 95% CI = 1.09–1.87) were associated with reduced renal function.

**Table 3:** Results of a multilevel logistic model to investigate the association between urbanization and reduced renal function among Chinese women (2009)

	Model 1			Model 2			Model 3			Model 4		
	OR	95% CI	*p*-value	OR	95% CI	*p*-value	OR	95% CI	*p*-value	OR	95% CI	*p*-value
Urbanization^a^	1.35	1.11, 1.62	0.002	1.29	1.06, 1.58	0.011	1.29	1.06, 1.57	0.012	1.24	1.01, 1.52	0.041
Health-related behavior												
Alcohol consumption^b^				0.75	0.42, 1.35	0.333	0.76	0.42, 1.36	0.349	0.74	0.41, 1.35	0.332
Total physical activity^c^				0.99	0.98, 1.00	0.296	0.99	0.98, 1.00	0.268	1.00	0.99, 1.01	0.444
Energy intake^d^				0.91	0.61, 1.36	0.640	0.82	0.56, 1.22	0.335	0.92	0.61, 1.39	0.699
Protein intake^e^				1.99	0.62, 6.44	0.250	0.95	0.33, 2.76	0.924	1.85	0.56, 6.10	0.311
Sodium intake				0.98	0.92, 1.04	0.452				0.97	0.92, 1.03	0.382
Potassium intake				0.64	0.45, 0.92	0.017				0.64	0.44, 0.92	0.016
Sodium-to-potassium ratio							0.98	0.92, 1.05	0.624			
Cardiometabolic risk factors												
Overweight/Obesity^f^										0.89	0.67, 1.18	0.424
Hypertension^g^										1.34	1.01, 1.78	0.039
Diabetes Mellitus^h^										2.07	1.47, 2.92	0.000
High LDL^i^										1.42	1.09, 1.87	0.010

Models were adjusted for age (in years), educational attainment (primary education or less, junior high school, high school and attained further education) and household income

^a^Results are expressed per one-SD increase

^b^The referent was those who drank less than once per month

^c^Results are expressed per 10 METs increase

^d^Results are expressed per 1000 kcal increase

^e^Results are expressed per 100 g increase

^f^The referent was those whose body mass index was 24.0 or less

^g^Hypertension was defined by either systolic pressure ≥ 140 mmHg, diastolic pressure ≥ 90 mmHg or self-reported antihypertensive medication

^h^Diabetes were defined by either hemoglobin A1c ≥ 6.5% or fasting blood glucose ≥126 mg/dL or self-reported diagnosis of DM

^i^Higher LDL was defined by LDL ≥ 130 mg/dL

Table 4 shows the results of multilevel logistic regression analyses using the 12 components of the urbanization index as explanatory variables. Among both sexes, the housing component was positively associated with higher odds of reduced renal function (men: OR = 1.51, 95% CI = 1.01–2.28; women: OR = 1.39, 95% CI = 1.01–1.93). In addition, among women, the traditional market component was also positively associated with reduced renal function (OR = 1.27, 95% CI = 1.01–1.58) while communication component (OR = 0.75, 95% CI = 0.59–0.95) and health component (OR = 0.80, 95% CI = 0.66–0.98) were rather inversely associated with reduced renal function.

**Table 4:** Results of multilevel logistic regression analyses using the 12 components of the urbanization index among Chinese participants (2009)

	Male participants			Female participants		
Urbanization components^a^	OR	95% CI	*p*-value	OR	95% CI	*p*-value
Population Density	1.13	0.88, 1.46	0.330	1.07	0.87, 1.32	0.498
Economic Component	0.90	0.64, 1.27	0.551	0.99	0.75, 1.31	0.960
Traditional Market	1.21	0.93, 1.58	0.161	1.27	1.01, 1.58	0.036
Modern Market	1.08	0.80, 1.47	0.606	1.23	0.95, 1.59	0.117
Transportation	0.90	0.71, 1.15	0.398	1.09	0.90, 1.32	0.391
Sanitation	0.90	0.61, 1.32	0.576	0.83	0.60, 1.15	0.272
Communications	0.85	0.63, 1.13	0.262	0.75	0.59, 0.95	0.017
Housing	1.51	1.01, 2.28	0.047	1.39	1.01, 1.93	0.046
Education	1.05	0.77, 1.42	0.762	1.17	0.90, 1.52	0.252
Diversity	0.84	0.64, 1.10	0.203	0.81	0.64, 1.01	0.064
Health	0.87	0.69, 1.11	0.261	0.80	0.66, 0.98	0.031
Social Service	1.16	0.92, 1.48	0.217	1.04	0.85, 1.28	0.689

Models were adjusted for age (in years), educational attainment (primary education or less, junior high school, high school and attained further education), household income, health-related behavior and cardiometabolic risk factors

^a^Results are expressed per one-SD increase

## Discussion

### Findings of this study

The prevalence of reduced renal function was 8.1% among CHNS participants aged 18 to 94 years of age. Furthermore, using a validated, multicomponent urbanization index, we found a positive association between urbanization and reduced renal function in the CHNS. Specifically, a one-standard deviation increase in the urbanization index was associated with a 1.38 (95%CI = 1.11–1.73) and 1.35 (95% CI = 1.11–1.62) times higher odds of reduced renal function among men and women, respectively. The associations remained statistically significant after adjusting for diet, physical activity, BMI and cardiometabolic risk factors only among women.

### Comparison with the previous studies in China: CKD prevalence

Reduced eGFR was observed among 8.1% of the participants. This is comparatively higher than that reported in previous studies in Chinese adults that used eGFR of less than 60 mL/min/1.73 m^2^ to indicate reduced renal function (i.e., 1.7 to 3.2%) [9, 14, 16, 40, 41]. For example, Zhang et al. [14] reported that the prevalence was 1.7%, using a nationally representative sample of Chinese adults (*n* = 47,204; mean age = 49.6 years) conducted in 2009 and 2010, and Chen et al. [9] reported it to be 3.2% among 6311 residents in Guangzhou (mean age = 51.6 years). However, when we calculated the prevalence among participants aged 40 years or older for comparability, the prevalence of reduced renal function (10.6%) fell between the ranges of those reported in previous research studying middle-aged and old people. For example, using China Health and Retirement Longitudinal Survey data in 2011 and 2012, Wang et al. [23] showed that reduced eGFR was reported among 11.5% of the participants. Zhang et al. [10] reported the prevalence of reduced renal function among those who lived in Beijing and were aged 40 years or older to be 5.2%. Chen et al. [42] showed the prevalence of reduced eGFR to be 2.5% among Chinese participants aged 35 to 74 years in 2000 to 2004. Li et al. [11] studied 2310 middle- and old-aged adults in Beijing and reported CKD and reduced eGFR in 12.9 and 4.9% of the population, respectively. Shan et al. [43] studied CKD in 4156 participants aged 40 years or older living in four major cities in Henan, finding reduced eGFR in 1.53% of the participants. The prevalence of reduced renal function we report in this study was somewhat high but still within the reported range.

### The association between community-level variables with CKD

We found that living in a more urbanized community were associated with higher odds of having reduced renal function. This is consistent with Wang et al. [23] showing that reduced eGFR prevalence was higher in urban (13.0%) compared with rural (10.0%) Chinese adults. However, other studies have not reported such an association [42] perhaps because these other studies were conducted earlier in the process of urbanization when its impact on reduced renal function might have been yet to appear. For example, using data from 15,540 Chinese general population aged 35 to 74 years collected in 2000 and 2001, Chen et al. [42] showed that there was no statistically significant difference in CKD prevalence between those who lived in urban (2.6%) and rural (2.5%) areas. Second, as previously mentioned, the crude, dichotomous classification of places as urban or rural might have blurred the association between urbanization and CKD. Zhang et al. [14] showed that the degree of economic development (operationalized with gross domestic product tertiles) was positively associated with albuminuria (one of the indicators of renal damage) only in rural communities. This finding may stem from the fact that among rural communities, urbanization is more straightforward to measure and could be evaluated with gross economic product only. In urban areas, economic development may need to be classified using a more nuanced approach than used in the Zhang study.

After adjusting for lifestyle-related factors and cardiometabolic risk factors (i.e., hypertension, DM and high LDL cholesterol), the association between urbanization and the odds of reduced renal function was attenuated in both sexes, which suggests that these risk factors underlie the association between urbanization and CKD. However, given that the association between urbanization and reduced renal function remained statistically significant particularly in women, other aspects of living in more urbanized environments [6, 44] may be associated with reduced renal function, particularly in women.

In this context, the analysis incorporating the 12 components of the urbanization index may help identify the aspects which were associated with reduced renal function. The associations observed in the analysis suggest that the impact of urbanization on renal function might vary depending on aspects of urbanization we focus on. As most of the information included in each component did not seem to have direct effects on renal function [25], the associations observed in this study might have been due to some other factors associated with the aspect of urbanization process. For example, the housing component consists of information on availability of electricity, indoor tap water, flush toilet and cooking gas while the traditional market component consists of information on distance to the market, the number of days of operation for eight types of market, none of which have been previously shown to be linked directly with chronic kidney disease; the two components which were found to be associated with lower odds of reduced renal function in women also did not seem to directly affect renal function. However, future research can pay attention to these aspects of urbanization to elucidate exact mechanisms linking urbanization and reduced renal function while these single elements of urbanization may not fully represent the sum total effects of urbanization.

### The association between individual-level variables and CKD

Alcohol consumption was inversely associated with the prevalence of reduced renal function in this study; reverse causality might be the case (i.e., old participants who had reduced renal function were also the ones who did not drink alcohol). In contrast, other researchers have found a positive association between alcohol consumption and CKD risk [19], while others have reported an inverse association [45–47]. Although health risks associated with alcohol consumption are well recognized [48], alcohol consumption might be protective for renal function. While some studies [9, 10, 17–19] suggested a higher risk of CKD among smokers with some exceptions [16], we did not find a statistically significant difference between smokers and non-smokers. While this insignificance might have been due to lack of information on past smoking status, future study is needed to account for second-hand smoking, as more than half of men in China are smokers, which might influence others irrespective of their own smoking status. Evidence suggests that second hand smoking affects proteinuria in children with CKD [49].

Those men who engaged in higher physical activity had lower odds of reduced renal function, but this was attenuated after adjusting for cardiometabolic risk factors. As previous studies also showed an association between physical inactivity and CKD [17, 18], physical activity may influence renal function through cardiometabolic risk factors. We did not find a statistical association between sodium intake and reduced renal function in this study. A systematic review by Smyth et al. [50] reported conflicting results in relation to sodium intake and suggested that both high intake and low intake may associate with higher risk of adverse renal outcomes compare with moderate intake; we did not find such an association when we used a squared term of sodium intake (data not shown). While there is limited study on the association between dietary potassium and reduced renal function, research on other health outcomes has produced similar findings to the association observed in women in our study; Khaw et al. [51] reported that low potassium intake was associated with higher risk of stroke-associated mortality in women (relative risk [RR] = 4.8, *p* = 0.01) and that this association was less pronounced in men (RR = 2.6, *p* = 0.16).

### Strengths and limitations

There are some limitations to this study. First, we did not have information on albuminuria as we did not collect urinary samples. Albuminuria is used to diagnose CKD in clinical settings and this could have led us to fail to identify some of those who had CKD. Also, creatinine concentrations were only measured once for each participant while CKD is generally defined as the presence of either kidney damage or decreased kidney function for more than three months [34]. Second, the cross-sectional design of the study prevented us from making causal inference. This might be especially important in relation to the association of alcohol consumption, total physical activity, and potassium intake with reduced renal function, which were shown to be associated with reduced renal function but which also could have been due to reverse causality. In addition, the effect of smoking status was investigated only based on current smoking status in this study. This might have been problematic as cadmium, one of the important sources of which has been reported to be smoking in China, may have accumulated throughout one’s life and eventually caused renal damage [52].

Despite the limitations described above, this study has strengths which extend the previous studies on the renal function in the developing countries. Specifically, we found that the prevalence of reduced renal function increased along with urbanization, independent of other cardiometabolic risk factors. This should be taken into consideration by public health officers and researchers working in developing countries where economic development and urbanization are rapidly taking place. Future studies are needed to identify biological pathways, which may link urbanization and the occurrence of reduced renal function. It should also be mentioned that our multidimensional measure of urbanization captures heterogeneity in a variety of services and infrastructure within and between high- and low-urbanized areas. When we decomposed urbanization into its components we saw the significant associations with specific elements of urbanization, but these single associations may not have fully represented the sum total effects of urbanization on renal function.

## Conclusion

In Chinese adults, we found that living in an urban environment was associated with higher odds of reduced renal function, independently of behavioral and cardiometabolic risk factors which have also been shown to increase along with urbanization process.

## Additional file

Additional file 1: Table S1. Characteristics of the study participants in China Health and Nutrition Survey (2009), stratified by the degree of urbanization. It contains basic characteristics of the study participants, stratified by the degree of urbanization. (DOCX 25 kb)

## Abbreviations

BMI: Body mass index
CHNS: China Health and Nutrition Survey
CKD: Chronic kidney disease
CKD-EPI: Chronic kidney disease-epidemiology equation
DM: Diabetes mellitus
eGFR: Estimated glomerular filtration rate
LDL: Low-density lipoprotein cholesterol
MET: Metabolic equivalent
OR: Odds ratio
RR: Relative risk
Scr: Serum creatinine concentration
SD: Standard deviation
SES: Socioeconomic status
