# Supplementary material for: The association between urbanization and reduced renal function: findings from the China Health and Nutrition Survey
Source: BMC Nephrol. 2017 May 15;18:160. doi: 10.1186/s12882-017-0577-7 (PMC5433002; doi:10.1186/s12882-017-0577-7)
Supplement: Additional file 1: Table S1. — Characteristics of the study participants in China Health and Nutrition Survey (2009), stratified by the degree of urbanization. It contains basic characteristics of the study participants, stratified by the degree of urbanization. (DOCX 25 kb) [file 12882_2017_577_MOESM1_ESM.docx]

Additional file 1: Table S1. Basic characteristics of the study participants in China Health and Nutrition Survey (2009), stratified by the degree of urbanization

|  | Urbanization index | | | | | | | | | p-value^a^ | |
| --- | --- | --- | --- | --- | --- | --- | --- | --- | --- | --- | --- |
|  | 1st Quartile  (n = 1969) | | 2nd Quartile  (n = 1962) | | 3rd Quartile  (n = 1938) | | 4th Quartile  (n = 1929) | | |  |  |
| Age (in years), mean [SD] | 50.3 | 14.9 | 50.2 | 14.5 | 50.5 | 14.9 | 52.8 | 15.3 | < 0.001 | |  |
| Sex (% female) | 1012 | 51.4 | 1070 | 54.5 | 1024 | 52.8 | 1048 | 54.3 | 0.168 | |  |
| Education, n (%) |  |  |  |  |  |  |  |  |  | |  |
| Primary school or less | 1184 | 60.1 | 1040 | 53.0 | 712 | 36.7 | 481 | 24.9 | < 0.001 | |  |
| Junior high school | 624 | 31.7 | 707 | 36.0 | 665 | 34.3 | 570 | 29.6 |  | |  |
| Senior high school | 108 | 5.5 | 145 | 7.4 | 300 | 16.1 | 329 | 17.1 |  | |  |
| Post-secondary education | 53 | 2.7 | 70 | 3.6 | 250 | 12.9 | 549 | 28.5 |  | |  |
| Household income, n (%) |  |  |  |  |  |  |  |  |  | |  |
| Low (0 – 6,533) | 831 | 42.2 | 707 | 36.0 | 661 | 34.1 | 402 | 20.8 | < 0.001 | |  |
| Middle (6,542 – 13,859) | 700 | 35.6 | 705 | 35.9 | 634 | 32.7 | 559 | 29.0 |  | |  |
| High (13,862 – 378,571) | 438 | 22.2 | 550 | 28.0 | 643 | 33.2 | 968 | 50.2 |  | |  |
| Health-related behavior |  |  |  |  |  |  |  |  |  | |  |
| Alcohol consumption frequency, n (%) | 585 | 29.7 | 569 | 29.0 | 545 | 28.1 | 494 | 25.6 | 0.026 | |  |
| Current Smoking, n (%) | 653 | 33.2 | 543 | 27.7 | 512 | 26.4 | 466 | 24.2 | < 0.001 | |  |
| Weekly physical activity (METs), mean [SD] | 316.3 | 262.7 | 276.1 | 228.4 | 158.7 | 154.1 | 112.9 | 95.4 | < 0.001 | |  |
| Energy intake (kcal), mean [SD] | 2192.3 | 615.6 | 2217.0 | 623.1 | 2100.5 | 568.6 | 2030.8 | 563.9 | < 0.001 | |  |
| Protein intake (g), mean [SD] | 62.3 | 20.7 | 66.3 | 22.2 | 66.9 | 22.1 | 67.9 | 22.1 | < 0.001 | |  |
| Sodium intake (g), mean [SD] | 4.8 | 2.7 | 4.7 | 2.7 | 4.6 | 23.5 | 4.6 | 2.6 | 0.283 | |  |
| Potassium intake (g), mean [SD] | 1.7 | 0.6 | 1.6 | 0.6 | 1.6 | 0.6 | 1.7 | 0.6 | < 0.001 | |  |
| Sodium-to-potassium ratio, mean [SD] | 3.1 | 2.0 | 3.2 | 2.1 | 3.1 | 2.0 | 3.0 | 1.9 | < 0.001 | |  |
| Cardiometabolic risk factors |  |  |  |  |  |  |  |  |  | |  |
| Body mass index, n (%) |  |  |  |  |  |  |  |  |  | |  |
| < 24.0 | 1290 | 65.5 | 1172 | 59.7 | 1083 | 55.9 | 1122 | 58.2 | < 0.001 | |  |
| 24.0 - 27.99 | 523 | 26.6 | 616 | 31.4 | 626 | 32.3 | 607 | 31.5 |  | |  |
| ≥ 28.0 | 156 | 7.9 | 174 | 8.9 | 229 | 11.8 | 200 | 10.4 |  | |  |
| Hypertension, n (%) | 566 | 28.8 | 596 | 30.4 | 594 | 30.7 | 668 | 34.6 | 0.001 | |  |
| Diabetes mellitus, n (%) | 159 | 8.1 | 186 | 9.5 | 265 | 13.7 | 258 | 13.4 | < 0.001 | |  |
| High LDL, n (%) | 463 | 23.5 | 563 | 28.7 | 686 | 35.4 | 676 | 35.0 | < 0.001 | |  |
| Urbanization index, mean [SD] | 42.9 | 5.3 | 56.8 | 3.6 | 76.6 | 6.8 | 92.3 | 4.5 | - | |  |
| eGFR, mean [SD] | 84.8 | 21.4 | 82.8 | 15.5 | 80.9 | 16.9 | 77.8 | 16.1 | < 0.001 | |  |

^a^ Characteristics of the participants were compared using t-test for continuous variables and χ2 test for categorical variables.
